# 4-(Dimethyl­amino)­pyridinium tetra­chlorido(pyridine-2-carboxyl­ato-κ^2^ *N*,*O*)stannate(IV)

**DOI:** 10.1107/S160053681201968X

**Published:** 2012-05-05

**Authors:** Ezzatollah Najafi, Mostafa M. Amini, Seik Weng Ng

**Affiliations:** a Department of Chemistry, General Campus, Shahid Beheshti University, Tehran 1983963113, Iran; b Department of Chemistry, University of Malaya, 50603 Kuala Lumpur, Malaysia; c Chemistry Department, Faculty of Science, King Abdulaziz University, PO Box 80203 Jeddah, Saudi Arabia

## Abstract

The reaction of 4-(dimethyl­amino)­pyridine, picolinic acid and stannic chloride yields the title salt, (C_7_H_11_N_2_)[SnCl_4_(C_6_H_4_NO_2_)], in which the Sn^IV^ atom is *N*,*O*-chelated by the picolinate ion in a *cis*-SnNOCl_4_ octa­hedral geometry. The cation is linked to the anion by an N—H⋯O hydrogen bond.

## Related literature

For 4-(dimethyl­amino)­pyridinium tetra­chlorido(quinoline-2-carboxyl­ato)stannate, see: Najafi *et al.* (2011 ▶).
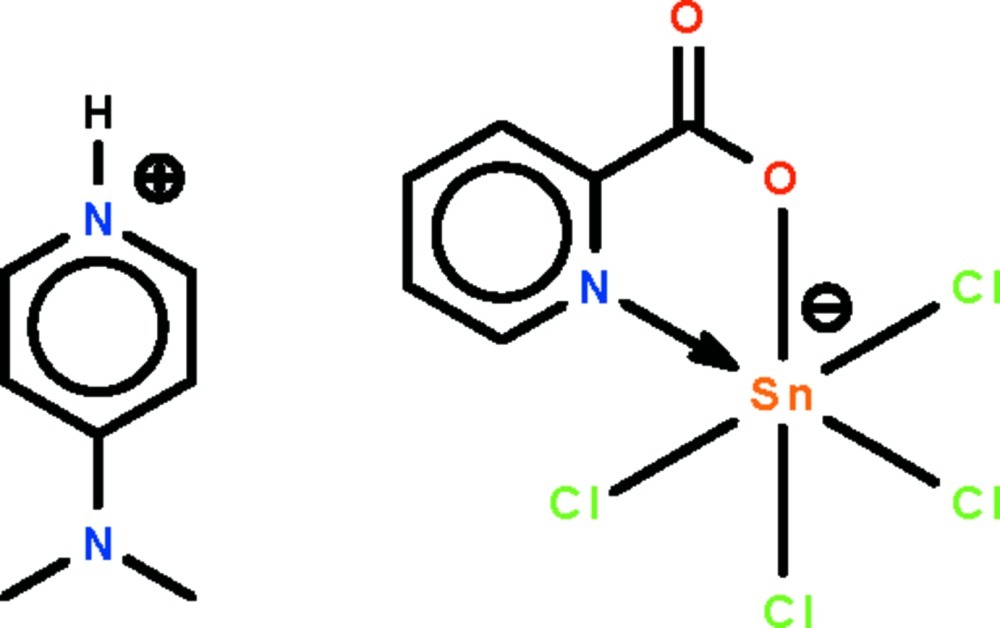


## Experimental

### 

#### Crystal data

(C_7_H_11_N_2_)[SnCl_4_(C_6_H_4_NO_2_)]
*M* *_r_* = 505.77
Triclinic, 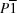

*a* = 7.6658 (2) Å
*b* = 9.8948 (4) Å
*c* = 13.5722 (6) Å
α = 69.485 (4)°
β = 83.159 (3)°
γ = 67.900 (3)°
*V* = 893.25 (6) Å^3^
*Z* = 2
Mo *K*α radiation
μ = 2.04 mm^−1^
*T* = 100 K
0.30 × 0.25 × 0.20 mm

#### Data collection

Agilent SuperNova Dual diffractometer with an Atlas detector
Absorption correction: multi-scan (*CrysAlis PRO*; Agilent, 2012 ▶) *T*_min_ = 0.580, *T*_max_ = 0.686
12966 measured reflections
4109 independent reflections
3744 reflections with *I* > 2σ(*I*)
*R*_int_ = 0.029

#### Refinement

*R*[*F* ^2^ > 2σ(*F* ^2^)] = 0.020
*wR*(*F* ^2^) = 0.049
*S* = 1.02
4109 reflections
214 parameters
1 restraint
H atoms treated by a mixture of independent and constrained refinement
Δρ_max_ = 0.61 e Å^−3^
Δρ_min_ = −0.41 e Å^−3^

### 

Data collection: *CrysAlis PRO* (Agilent, 2012 ▶); cell refinement: *CrysAlis PRO*; data reduction: *CrysAlis PRO*; program(s) used to solve structure: *SHELXS97* (Sheldrick, 2008 ▶); program(s) used to refine structure: *SHELXL97* (Sheldrick, 2008 ▶); molecular graphics: *X-SEED* (Barbour, 2001 ▶); software used to prepare material for publication: *publCIF* (Westrip, 2010 ▶).

## Supplementary Material

Crystal structure: contains datablock(s) global, I. DOI: 10.1107/S160053681201968X/bt5909sup1.cif

Structure factors: contains datablock(s) I. DOI: 10.1107/S160053681201968X/bt5909Isup2.hkl

Additional supplementary materials:  crystallographic information; 3D view; checkCIF report

## Figures and Tables

**Table 1:** Hydrogen-bond geometry (Å, °)

*D*—H⋯*A*	*D*—H	H⋯*A*	*D*⋯*A*	*D*—H⋯*A*
N2—H2⋯O1	0.87 (1)	1.93 (1)	2.802 (2)	176 (2)

**Fig. 1.**
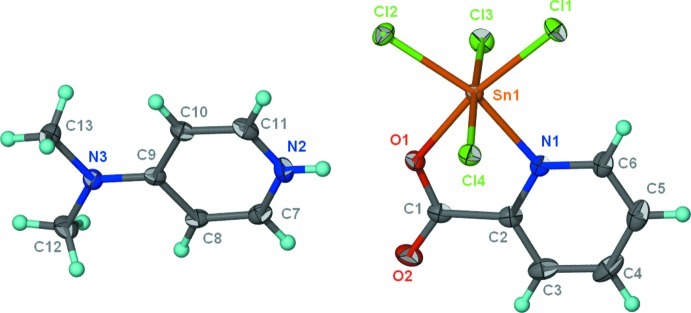
Anisotropic displacement ellipsoid plot (Barbour, 2001) of (C7H11N2)[SnCl4(C6H4NO2)] at the 70% probability level; hydrogen atoms are drawn as spheres of arbitrary radius.

## supplementary crystallographic information

### Comment

A previous study reported 4-(dimethylamino)pyridinium tetrachlorido(quinoline-2-carboxylato)stannate, which was synthesized by the reaction of 4-(dimethylamino)pyridine, quinoline-2-carboxylic acid and stannic chloride in methanol (Najafi *et al.*, 2011). The reaction with picolinic acid in place of quinoline-2-carboxylic acid yielded the analogous salt, (C_7_H_11_N_2_)[SnCl_4_(C_6_H_4_NO_2_)] (Scheme I). The Sn^IV^ atom is *N*,*O*-chelated by the picolinate ion in a *cis*-SnNOCl_4_ octahedral geometry (Fig. 1). The cation is linked to the anion by an N–H···O hydrogen bond (Table 1).

### Experimental

Stannic chloride pentahydrate (0.35 g, 1 mmol), picolinic acid (0.12 g, 1 mmol) and 4-(dimethylamino)pyridine (0.12 g, 1 mmol) were loaded into a convection tube; the tube was filled with dry methanol and kept at 333 K. Colorless crystals were collected from the side arm after several days.

### Refinement

Carbon-bound H-atoms were placed in calculated positions [C–H 0.95 to 0.98 Å, *U*_iso_(H) 1.2 to 1.5*U*_eq_(C)] and were included in the refinement in the riding model approximation.

The pyridinium H-atom was located in a difference Fourier map, and was refined isotropically with a distance restraint of N–H 0.88±0.01 Å.

### Crystal data

**Table:**

(C_7_H_11_N_2_)[SnCl_4_(C_6_H_4_NO_2_)]	*Z* = 2
*M**_r_* = 505.77	*F*(000) = 496
Triclinic, *P*1	*D*_x_ = 1.880 Mg m^−^^3^
Hall symbol: -P 1	Mo *K*α radiation, λ = 0.71073 Å
*a* = 7.6658 (2) Å	Cell parameters from 8614 reflections
*b* = 9.8948 (4) Å	θ = 2.4–27.5°
*c* = 13.5722 (6) Å	µ = 2.04 mm^−^^1^
α = 69.485 (4)°	*T* = 100 K
β = 83.159 (3)°	Prism, colorless
γ = 67.900 (3)°	0.30 × 0.25 × 0.20 mm
*V* = 893.25 (6) Å^3^	

### Data collection

**Table:**

Agilent SuperNova Dual diffractometer with an Atlas detector	4109 independent reflections
Radiation source: SuperNova (Mo) X-ray Source	3744 reflections with *I* > 2σ(*I*)
Mirror monochromator	*R*_int_ = 0.029
Detector resolution: 10.4041 pixels mm^-1^	θ_max_ = 27.6°, θ_min_ = 2.4°
ω scan	*h* = −9→9
Absorption correction: multi-scan (*CrysAlis PRO*; Agilent, 2012)	*k* = −12→12
*T*_min_ = 0.580, *T*_max_ = 0.686	*l* = −17→17
12966 measured reflections	

### Refinement

**Table:**

Refinement on *F*^2^	Primary atom site location: structure-invariant direct methods
Least-squares matrix: full	Secondary atom site location: difference Fourier map
*R*[*F*^2^ > 2σ(*F*^2^)] = 0.020	Hydrogen site location: inferred from neighbouring sites
*wR*(*F*^2^) = 0.049	H atoms treated by a mixture of independent and constrained refinement
*S* = 1.02	*w* = 1/[σ^2^(*F*_o_^2^) + (0.0217*P*)^2^ + 0.1127*P*] where *P* = (*F*_o_^2^ + 2*F*_c_^2^)/3
4109 reflections	(Δ/σ)_max_ = 0.002
214 parameters	Δρ_max_ = 0.61 e Å^−^^3^
1 restraint	Δρ_min_ = −0.41 e Å^−^^3^

### Fractional atomic coordinates and isotropic or equivalent isotropic displacement parameters (Å^2^)

**Table:**

	*x*	*y*	*z*	*U*_iso_*/*U*_eq_	
Sn1	0.471694 (16)	0.931155 (14)	0.787934 (10)	0.01225 (5)	
Cl1	0.41910 (7)	1.16043 (5)	0.82535 (4)	0.01887 (11)	
Cl2	0.63070 (7)	0.97801 (6)	0.62519 (4)	0.01884 (11)	
Cl3	0.76267 (6)	0.79583 (6)	0.88640 (4)	0.01957 (11)	
Cl4	0.16639 (6)	1.04910 (5)	0.70014 (4)	0.01687 (10)	
O1	0.48413 (18)	0.72139 (15)	0.77597 (11)	0.0164 (3)	
O2	0.3917 (2)	0.51987 (16)	0.84279 (12)	0.0231 (3)	
N1	0.3269 (2)	0.83176 (18)	0.93069 (12)	0.0137 (3)	
N2	0.6230 (2)	0.5870 (2)	0.61853 (14)	0.0184 (4)	
H2	0.574 (3)	0.630 (3)	0.6665 (15)	0.038 (7)*	
N3	0.8688 (2)	0.35910 (18)	0.40725 (13)	0.0153 (3)	
C1	0.3977 (3)	0.6389 (2)	0.84580 (16)	0.0159 (4)	
C2	0.3057 (3)	0.7014 (2)	0.93305 (15)	0.0155 (4)	
C3	0.2055 (3)	0.6298 (2)	1.01141 (17)	0.0217 (5)	
H3	0.1910	0.5382	1.0122	0.026*	
C4	0.1268 (3)	0.6952 (3)	1.08887 (17)	0.0270 (5)	
H4	0.0545	0.6500	1.1428	0.032*	
C5	0.1535 (3)	0.8254 (3)	1.08742 (16)	0.0235 (5)	
H5	0.1024	0.8694	1.1412	0.028*	
C6	0.2552 (3)	0.8922 (2)	1.00731 (15)	0.0178 (4)	
H6	0.2744	0.9819	1.0066	0.021*	
C7	0.6389 (3)	0.4385 (2)	0.64189 (16)	0.0176 (4)	
H7	0.5927	0.3883	0.7070	0.021*	
C8	0.7198 (2)	0.3592 (2)	0.57414 (15)	0.0153 (4)	
H8	0.7304	0.2546	0.5922	0.018*	
C9	0.7888 (2)	0.4335 (2)	0.47584 (15)	0.0137 (4)	
C10	0.7676 (3)	0.5896 (2)	0.45468 (16)	0.0159 (4)	
H10	0.8102	0.6444	0.3900	0.019*	
C11	0.6865 (3)	0.6614 (2)	0.52659 (17)	0.0183 (4)	
H11	0.6745	0.7657	0.5117	0.022*	
C12	0.8845 (3)	0.1993 (2)	0.42939 (17)	0.0213 (5)	
H12A	0.9601	0.1329	0.4937	0.032*	
H12B	0.9454	0.1647	0.3704	0.032*	
H12C	0.7585	0.1933	0.4390	0.032*	
C13	0.9392 (3)	0.4362 (2)	0.30663 (16)	0.0201 (4)	
H13A	1.0157	0.4893	0.3186	0.030*	
H13B	0.8327	0.5118	0.2599	0.030*	
H13C	1.0164	0.3592	0.2741	0.030*	

### Atomic displacement parameters (Å^2^)

**Table:**

	*U*^11^	*U*^22^	*U*^33^	*U*^12^	*U*^13^	*U*^23^
Sn1	0.01423 (8)	0.01017 (8)	0.01266 (8)	−0.00471 (5)	0.00094 (5)	−0.00403 (5)
Cl1	0.0237 (2)	0.0125 (2)	0.0218 (3)	−0.00547 (19)	−0.00284 (19)	−0.0074 (2)
Cl2	0.0212 (2)	0.0168 (2)	0.0171 (3)	−0.00816 (19)	0.00602 (18)	−0.00436 (19)
Cl3	0.0165 (2)	0.0175 (2)	0.0221 (3)	−0.00282 (19)	−0.00390 (18)	−0.0058 (2)
Cl4	0.0166 (2)	0.0169 (2)	0.0167 (2)	−0.00586 (19)	−0.00196 (17)	−0.00459 (19)
O1	0.0222 (7)	0.0122 (7)	0.0173 (7)	−0.0088 (6)	0.0041 (5)	−0.0062 (6)
O2	0.0294 (8)	0.0166 (8)	0.0270 (9)	−0.0130 (6)	0.0004 (6)	−0.0063 (6)
N1	0.0137 (8)	0.0129 (8)	0.0121 (8)	−0.0034 (6)	−0.0013 (6)	−0.0025 (7)
N2	0.0170 (8)	0.0200 (9)	0.0193 (9)	−0.0035 (7)	−0.0013 (7)	−0.0105 (8)
N3	0.0178 (8)	0.0118 (8)	0.0167 (9)	−0.0054 (7)	0.0020 (6)	−0.0054 (7)
C1	0.0149 (9)	0.0134 (10)	0.0178 (10)	−0.0052 (8)	−0.0041 (7)	−0.0019 (8)
C2	0.0136 (9)	0.0131 (10)	0.0156 (10)	−0.0032 (8)	−0.0040 (7)	−0.0001 (8)
C3	0.0189 (10)	0.0203 (11)	0.0214 (11)	−0.0093 (8)	−0.0026 (8)	0.0020 (9)
C4	0.0186 (10)	0.0331 (13)	0.0181 (12)	−0.0088 (9)	0.0027 (8)	0.0032 (10)
C5	0.0190 (10)	0.0286 (12)	0.0143 (11)	−0.0009 (9)	−0.0011 (8)	−0.0050 (9)
C6	0.0168 (10)	0.0197 (11)	0.0133 (10)	−0.0028 (8)	−0.0017 (7)	−0.0047 (8)
C7	0.0144 (9)	0.0201 (11)	0.0168 (10)	−0.0071 (8)	−0.0009 (7)	−0.0031 (8)
C8	0.0138 (9)	0.0125 (10)	0.0177 (10)	−0.0052 (8)	−0.0010 (7)	−0.0018 (8)
C9	0.0105 (9)	0.0130 (10)	0.0165 (10)	−0.0035 (7)	−0.0027 (7)	−0.0036 (8)
C10	0.0174 (9)	0.0120 (10)	0.0179 (10)	−0.0060 (8)	−0.0029 (7)	−0.0027 (8)
C11	0.0177 (10)	0.0120 (10)	0.0244 (11)	−0.0036 (8)	−0.0062 (8)	−0.0048 (8)
C12	0.0227 (10)	0.0138 (10)	0.0289 (12)	−0.0058 (8)	0.0011 (9)	−0.0099 (9)
C13	0.0208 (10)	0.0208 (11)	0.0170 (11)	−0.0067 (9)	0.0030 (8)	−0.0059 (9)

### Geometric parameters (Å, º)

**Table:**

Sn1—O1	2.1041 (13)	C4—C5	1.372 (3)
Sn1—N1	2.2194 (16)	C4—H4	0.9500
Sn1—Cl1	2.3743 (5)	C5—C6	1.381 (3)
Sn1—Cl2	2.3761 (5)	C5—H5	0.9500
Sn1—Cl3	2.4006 (5)	C6—H6	0.9500
Sn1—Cl4	2.4277 (5)	C7—C8	1.359 (3)
O1—C1	1.311 (2)	C7—H7	0.9500
O2—C1	1.210 (2)	C8—C9	1.427 (3)
N1—C6	1.339 (2)	C8—H8	0.9500
N1—C2	1.348 (3)	C9—C10	1.416 (3)
N2—C11	1.348 (3)	C10—C11	1.361 (3)
N2—C7	1.350 (3)	C10—H10	0.9500
N2—H2	0.874 (10)	C11—H11	0.9500
N3—C9	1.338 (2)	C12—H12A	0.9800
N3—C12	1.462 (3)	C12—H12B	0.9800
N3—C13	1.464 (2)	C12—H12C	0.9800
C1—C2	1.506 (3)	C13—H13A	0.9800
C2—C3	1.382 (3)	C13—H13B	0.9800
C3—C4	1.386 (3)	C13—H13C	0.9800
C3—H3	0.9500		
			
O1—Sn1—N1	75.84 (6)	C5—C4—H4	120.1
O1—Sn1—Cl1	169.89 (4)	C3—C4—H4	120.1
N1—Sn1—Cl1	94.27 (4)	C4—C5—C6	119.6 (2)
O1—Sn1—Cl2	88.75 (4)	C4—C5—H5	120.2
N1—Sn1—Cl2	164.57 (4)	C6—C5—H5	120.2
Cl1—Sn1—Cl2	101.090 (18)	N1—C6—C5	120.7 (2)
O1—Sn1—Cl3	89.31 (4)	N1—C6—H6	119.6
N1—Sn1—Cl3	88.57 (4)	C5—C6—H6	119.6
Cl1—Sn1—Cl3	92.581 (18)	N2—C7—C8	121.32 (19)
Cl2—Sn1—Cl3	92.190 (18)	N2—C7—H7	119.3
O1—Sn1—Cl4	87.20 (4)	C8—C7—H7	119.3
N1—Sn1—Cl4	86.38 (4)	C7—C8—C9	119.91 (19)
Cl1—Sn1—Cl4	90.124 (17)	C7—C8—H8	120.0
Cl2—Sn1—Cl4	92.055 (17)	C9—C8—H8	120.0
Cl3—Sn1—Cl4	174.439 (17)	N3—C9—C10	121.88 (18)
C1—O1—Sn1	119.22 (12)	N3—C9—C8	121.49 (18)
C6—N1—C2	120.02 (17)	C10—C9—C8	116.63 (18)
C6—N1—Sn1	126.65 (14)	C11—C10—C9	120.29 (19)
C2—N1—Sn1	113.24 (12)	C11—C10—H10	119.9
C11—N2—C7	120.66 (18)	C9—C10—H10	119.9
C11—N2—H2	122.3 (18)	N2—C11—C10	121.18 (19)
C7—N2—H2	117.0 (18)	N2—C11—H11	119.4
C9—N3—C12	120.98 (17)	C10—C11—H11	119.4
C9—N3—C13	121.09 (17)	N3—C12—H12A	109.5
C12—N3—C13	117.91 (16)	N3—C12—H12B	109.5
O2—C1—O1	123.64 (18)	H12A—C12—H12B	109.5
O2—C1—C2	121.19 (18)	N3—C12—H12C	109.5
O1—C1—C2	115.16 (17)	H12A—C12—H12C	109.5
N1—C2—C3	121.61 (19)	H12B—C12—H12C	109.5
N1—C2—C1	116.16 (17)	N3—C13—H13A	109.5
C3—C2—C1	122.22 (19)	N3—C13—H13B	109.5
C2—C3—C4	118.2 (2)	H13A—C13—H13B	109.5
C2—C3—H3	120.9	N3—C13—H13C	109.5
C4—C3—H3	120.9	H13A—C13—H13C	109.5
C5—C4—C3	119.8 (2)	H13B—C13—H13C	109.5
			
N1—Sn1—O1—C1	4.83 (13)	O1—C1—C2—N1	−1.6 (2)
Cl1—Sn1—O1—C1	−7.3 (3)	O2—C1—C2—C3	−2.2 (3)
Cl2—Sn1—O1—C1	−174.26 (13)	O1—C1—C2—C3	178.52 (17)
Cl3—Sn1—O1—C1	93.53 (13)	N1—C2—C3—C4	−0.3 (3)
Cl4—Sn1—O1—C1	−82.14 (13)	C1—C2—C3—C4	179.61 (17)
O1—Sn1—N1—C6	178.01 (16)	C2—C3—C4—C5	−1.5 (3)
Cl1—Sn1—N1—C6	−4.12 (15)	C3—C4—C5—C6	1.5 (3)
Cl2—Sn1—N1—C6	−178.59 (11)	C2—N1—C6—C5	−2.2 (3)
Cl3—Sn1—N1—C6	88.36 (15)	Sn1—N1—C6—C5	174.16 (13)
Cl4—Sn1—N1—C6	−93.97 (15)	C4—C5—C6—N1	0.4 (3)
O1—Sn1—N1—C2	−5.40 (12)	C11—N2—C7—C8	0.3 (3)
Cl1—Sn1—N1—C2	172.47 (12)	N2—C7—C8—C9	−0.4 (3)
Cl2—Sn1—N1—C2	−2.0 (2)	C12—N3—C9—C10	−178.27 (17)
Cl3—Sn1—N1—C2	−95.05 (12)	C13—N3—C9—C10	0.2 (3)
Cl4—Sn1—N1—C2	82.62 (12)	C12—N3—C9—C8	1.5 (3)
Sn1—O1—C1—O2	177.24 (15)	C13—N3—C9—C8	−179.96 (16)
Sn1—O1—C1—C2	−3.5 (2)	C7—C8—C9—N3	−179.87 (17)
C6—N1—C2—C3	2.2 (3)	C7—C8—C9—C10	−0.1 (3)
Sn1—N1—C2—C3	−174.69 (14)	N3—C9—C10—C11	−179.60 (17)
C6—N1—C2—C1	−177.72 (16)	C8—C9—C10—C11	0.6 (3)
Sn1—N1—C2—C1	5.44 (19)	C7—N2—C11—C10	0.3 (3)
O2—C1—C2—N1	177.67 (17)	C9—C10—C11—N2	−0.7 (3)

### Hydrogen-bond geometry (Å, º)

**Table:**

*D*—H···*A*	*D*—H	H···*A*	*D*···*A*	*D*—H···*A*
N2—H2···O1	0.87 (1)	1.93 (1)	2.802 (2)	176 (2)
